# Urinary myo-inositol is associated with the clinical outcome in focal segmental glomerulosclerosis

**DOI:** 10.1038/s41598-019-51276-9

**Published:** 2019-10-11

**Authors:** Jung Nam An, Jin Seong Hyeon, Youngae Jung, Young Wook Choi, Jin Hyuk Kim, Seung Hee Yang, Sohee Oh, Soie Kwon, Sang-Ho Lee, Jang-Hee Cho, Sun-Hee Park, Hunjoo Ha, Dong Ki Kim, Jung Pyo Lee, Geum-Sook Hwang

**Affiliations:** 1 Department of Internal Medicine-Nephrology, Seoul National University Boramae Medical Center, Seoul, Korea; 2 Integrated Metabolomics Research Group, Western Seoul Center, Korea Basic Science Institute, Seoul, Korea; 3 Graduate School of Pharmaceutical Sciences, Ewha Womans University, Seoul, Korea; 4 Kidney Research Institute, Seoul National University, Seoul, Korea; 5 Department of Biostatistics, Seoul National University Boramae Medical Center, Seoul, Korea; 6 Department of Internal Medicine-Nephrology, Seoul National University Hospital, Seoul, Korea; 7 Department of Internal Medicine-Nephrology, Kyung Hee University Hospital at Gangdong, Seoul, Korea; 8 Department of Internal Medicine-Nephrology, Kyungpook National University Hospital, Daegu, Korea; 9 Department of Internal Medicine, Seoul National University College of Medicine, Seoul, Korea; 10 Department of Chemistry and Nano Science, Ewha Womans University, Seoul, Korea

**Keywords:** Metabolomics, Focal segmental glomerulosclerosis

## Abstract

Focal segmental glomerulosclerosis (FSGS) and minimal change disease (MCD) have similar initial histological findings; however, their prognoses are distinct. Therefore, it is of great importance to discriminate FSGS from MCD in the early phase of disease and predict clinical prognosis. A discovery set of 184 urine samples (61 healthy control, 80 MCD, and 43 FSGS) and a validation set of 61 urine samples (12 healthy control, 26 MCD, and 23 FSGS) were collected at the time of kidney biopsy. Metabolic profiles were examined using nuclear magnetic resonance spectroscopy. Of 70 urinary metabolites, myo-inositol was significantly higher in FSGS patients than in control patients (discovery set, 2.34-fold, P < 0.001; validation set, 2.35-fold, P = 0.008) and MCD patients (discovery set, 2.48-fold, P = 0.002; validation set, 1.69-fold, P = 0.042). Myo-inositol showed an inverse relationship with the initial estimated glomerular filtration rate (eGFR) and was associated with the plasma level of soluble urokinase-type plasminogen activator receptor in FSGS patients. Myo-inositol treatment ameliorated the decreased expression of ZO-1 and synaptopodin in an *in vitro* FSGS model, and as myo-inositol increased, myo-inositol oxygenase tissue expression decreased proportionally to eGFR. Furthermore, urinary myo-inositol exhibited an increase in the power to discriminate FSGS patients, and its addition could better predict the response to initial treatment. In conclusion, urinary myo-inositol may be an important indicator in the diagnosis and treatment of FSGS patients.

## Introduction

Focal segmental glomerulosclerosis (FSGS) accounts for one-third of adult nephrotic syndrome^1^ and is divided into primary and secondary FSGS depending on the etiology. Although several studies have shown that soluble urokinase plasminogen-type activator receptor (suPAR) levels increase in primary FSGS^2^, there are reports that it is associated with decreased renal function rather than FSGS itself. Therefore, the usefulness of suPAR as a biomarker in primary FSGS is controversial^3–5^.

FSGS can be diagnosed through renal biopsy by confirmation of focal and segmental mesangial collapse and sclerosis. Sometimes, however, the biopsy results of FSGS are confused with minimal change disease (MCD); MCD is diagnosed initially, but FSGS is often diagnosed in a subsequent biopsy. This finding suggests that the sclerotic lesions of FSGS were not detected at the initial examination or that these two diseases had the same spectra and showed similar histological findings at the beginning and then progressed from MCD to FSGS^6^. In fact, substantial evidence supports the fact that primary FSGS and MCD are different phenotypes of the same disease^7^.

However, the response to treatment and prognosis of these two diseases are very different. MCD responds well to corticosteroid treatment, and 90% of patients experience complete remission (CR) after 6 months of treatment^8^. On the other hand, only 30% of FSGS patients experience CR despite immunosuppressive therapy for 6–9 months or longer, and half of them will progress to end-stage renal disease (ESRD) within a few years^9,10^. Therefore, it is very important to clearly distinguish between the two disease types and to predict the therapeutic response and prognosis. Thus, in this study, we tried to find a method to distinguish between these two diseases using noninvasive, easily obtained and repeatable urine specimens because kidney biopsy, a conventional diagnostic method, is highly invasive and difficult to repeat.

Recently, several metabolomics studies have been performed to identify potential early biomarkers or monitor real-time kidney function in patients with various kidney diseases^11^. Using metabolomic profiling, we investigated and validated significant urinary metabolites to differentiate FSGS patients from MCD patients and associate these metabolites with several clinical parameters and clinical outcomes in FSGS patients. In addition, we performed an *in vitro* FSGS study and immunohistochemistry to verify the association with urinary metabolites.

## Results

### Baseline characteristics and demographics

Baseline characteristics and demographics assessed at the time of kidney biopsy are described in Table 1. Patients who were diagnosed with FSGS had a higher frequency of hypertension as a comorbid disease and showed significantly worse baseline renal function than those diagnosed with MCD. However, the levels of baseline proteinuria were higher in the MCD patients than in the FSGS patients. Male sex, age, diabetes, and blood pressure were not different between the two groups.

**Table 1:** Demographics and baseline characteristics.

	Control (n = 61)	MCD (n = 80)	FSGS (n = 43)	P-value
Male sex	32 (52.5)	50 (62.5)	28 (65.1)	0.347
Age (years)	46 (32, 61)	50 (38, 69)	55 (30, 67)	<0.001
Body mass index (kg/m^2^)	23.3 (20.9, 27.3)	25.1 (22.5, 27.9)	24.1 (22.6, 26.8)	0.176
Smoking history	11 (27.5)	20 (25.0)	12 (27.9)	0.634
Systolic blood pressure (mmHg)	128.0 (116.0, 140.0)	125.0 (115.0, 136.8)	129.0 (120.0, 141.0)	0.233
Diastolic blood pressure (mmHg)	80.0 (70.0, 85.0)	77.5 (70.3, 84.8)	78.0 (72.0, 90.0)	0.566
Blood urea nitrogen (mg/dL)	—	16.0 (12.0, 27.0)	24.0 (11.0, 36.0)	0.427
Serum creatinine (mg/dL)	0.82 (0.67, 0.93)	0.89 (0.72, 1.40)	1.26 (0.87, 2.14)	<0.001
Estimated glomerular filtration rate (mL/min/1.73 m^2^)	91.1 (79.3, 107.5)	88.4 (50.6, 112.8)	55.2 (30.5, 83.8)	<0.001
Urine protein/creatinine ratio (mg/mgCr)	—	9.62 (6.38, 14.71)	5.83 (3.32, 10.23)	<0.001
Hypertension	11 (18.0)	20 (25.0)	31 (72.1)	<0.001
Diabetes mellitus	2 (3.3)	8 (10.0)	7 (16.3)	0.075
Coronary artery disease	3 (4.9)	2 (2.5)	1 (2.3)	0.441
Cerebrovascular attack	4 (6.6)	0 (0.0)	1 (2.3)	0.019
Malignancy	—	4 (5.0)	6 (14.0)	0.083

The data are presented as the median (25^th^, 75^th^ percentiles) or as a number (percentage, %).

### Urinary metabolite profiling using nuclear magnetic resonance

**Figure 1:**
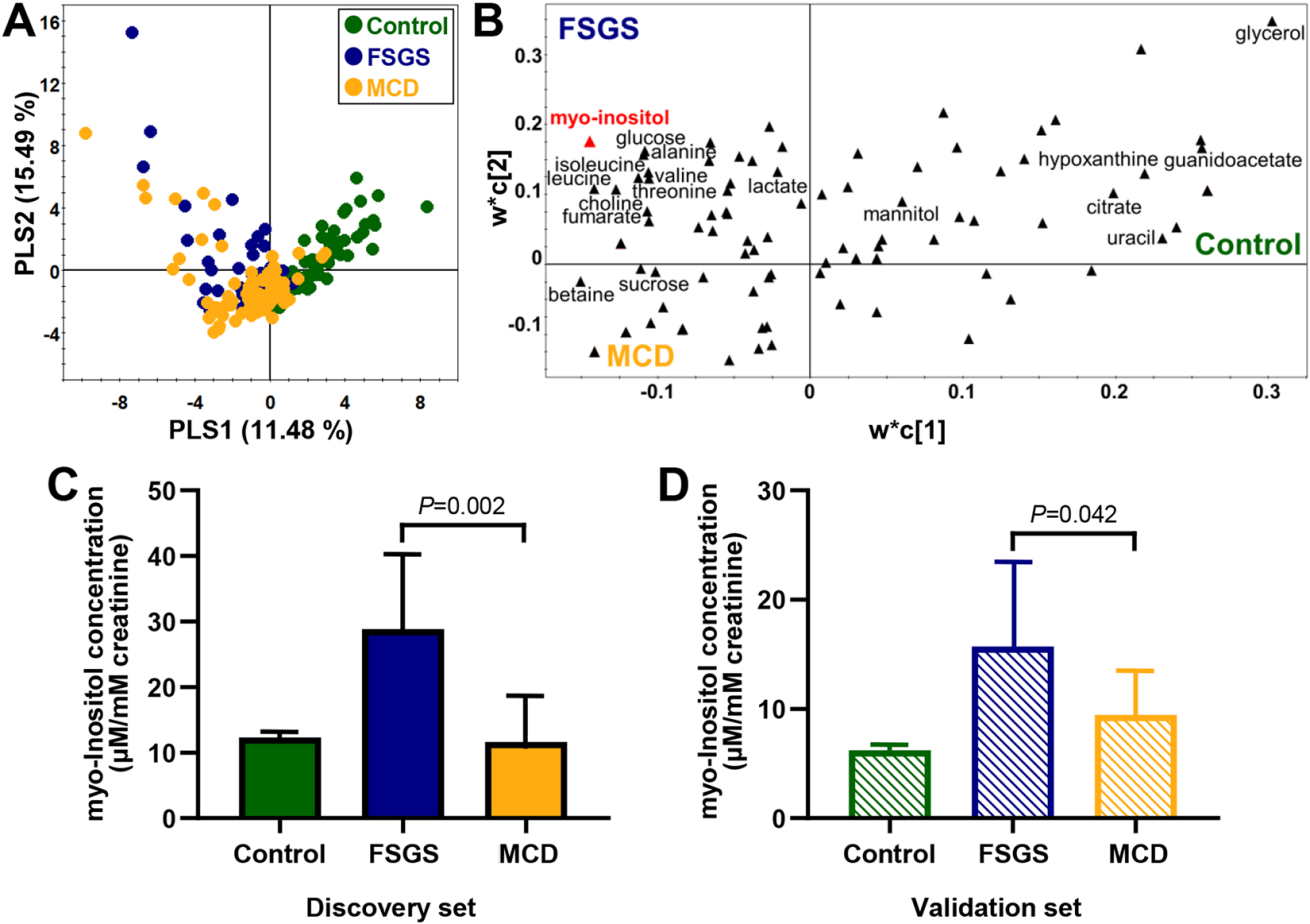
Multivariate statistical analyses of urine samples from FSGS, MCD and healthy controls. PLS-DA **(A)** score plot obtained from ^1^H NMR spectra of urine from FSGS, MCD, and healthy controls. The metabolites exhibiting significant differences in the urine are presented in the loading plot **(B)**. The log-transformed concentration of myo-inositol (μM/mM creatinine) in patients with FSGS was significantly altered compared with both healthy controls and MCD patients in the discovery set **(C)** and the validation set **(D)**. The data are shown as the median (*P < 0.05, **P < 0.01, ***P < 0.001, comparing healthy controls and FSGS).

Representative 800-MHz ^1^H nuclear magnetic resonance (NMR) spectra of urinary samples were obtained from FSGS patients, MCD patients and healthy controls (see Supplementary Fig. S1). Each NMR peak of 70 urinary metabolites was identified and quantified using the 800-MHz library within Chenomx software, two-dimensional (2D) NMR spectra (see Supplementary Figs S2, S3), and spiking experiments. Principal component analysis (PCA) was performed on 70 quantified urinary metabolites to assess the intrinsic variations among the FSGS, MCD and healthy control groups. The PCA did not show clear discrimination and outliers among the three groups (data not shown). We compared metabolic patterns between the three groups using a partial least squares discriminant analysis (PLS-DA) model derived from the quantified data. The PLS-DA score plot (Fig. 1A) showed a slight separation between the three groups with a goodness of fit of R^2^Y = 0.476 and predictability of Q^2^ = 0.298. Analysis of variance (ANOVA) of the cross-validated predictive residuals (CV-ANOVA) was used to assess the reliability of the PLS-DA models. The CV-ANOVA showed that the PLS-DA model was significant (P = 1.03E-20). The urinary metabolites exhibiting significant differences between groups are presented in the loading plot (Fig. 1B).

### Metabolic features in urine from FSGS patients

To identify specific metabolites associated with FSGS, we compared the concentrations of metabolites among FSGS, MCD and healthy control groups. The significantly different metabolites adjusted for age, sex, diabetes, and hypertension status between the FSGS and healthy control groups included cis-aconitate, trans-aconitate, alanine, betaine, choline, citrate, ethanolamine, fumarate, glucose, glycerol, guanidoacetate, histidine, 3-hydroxyisovalerate, hypoxanthine, 3-indoxylsulfate, isobutyrate, isoleucine, lactate, leucine, mannitol, methylamine, 1-methylnicotinamide, pyroglutamate, sucrose, threonine, tyrosine, uracil, valine, and myo-inositol (see Supplementary Table S1). In particular, myo-inositol concentrations were significantly higher in patients with FSGS than in both healthy controls (2.34-fold; P < 0.001) and MCD patients (2.48-fold; P = 0.002) (Fig. 1C); however, there was no difference between MCD patients and healthy controls. When the three groups were compared after matching for age, sex, and diabetes status, the significant differences in metabolites were almost unchanged from before matching (see Supplementary Table S2).

### Validation of identified urinary biomarkers

To validate whether myo-inositol is higher in patients with FSGS than in healthy controls and MCD patients, we recollected and analyzed an additional 61 urine samples (12 healthy controls, 26 MCD patients, and 23 primary FSGS patients). Baseline characteristics and demographics of the validation set are described in Supplementary Table S3. PCA (R^2^ = 0.276, Q^2^ = 0.014) was performed on 70 quantified urinary metabolites and did not show any outliers (see Supplementary Fig. S4A). The PLS-DA model (R^2^X = 0.18, R^2^Y = 0.454, Q^2^ = 0.205) showed a slight separation among the FSGS, MCD and healthy control groups (see Supplementary Fig. S4B). As displayed in the loading plot (see Supplementary Fig. S4C), myo-inositol was observed to be a metabolite with differences among the FSGS, MCD and healthy control groups. The significantly different metabolites adjusted for age, sex, diabetes, and hypertension status between FSGS patients and healthy control groups included cis-aconitate (0.49-fold; *P* = 0.006), creatine (0.43-fold; *P* = 0.017), histidine (0.44-fold; *P* = 0.027), and myo-inositol. The validation set confirmed that myo-inositol concentrations were significantly higher in patients with FSGS than in healthy controls (2.35-fold; P = 0.008) and MCD patients (1.69-fold; P = 0.042) (Fig. 1D).

### Association between urinary myo-inositol and clinical parameters

**Figure 2:**
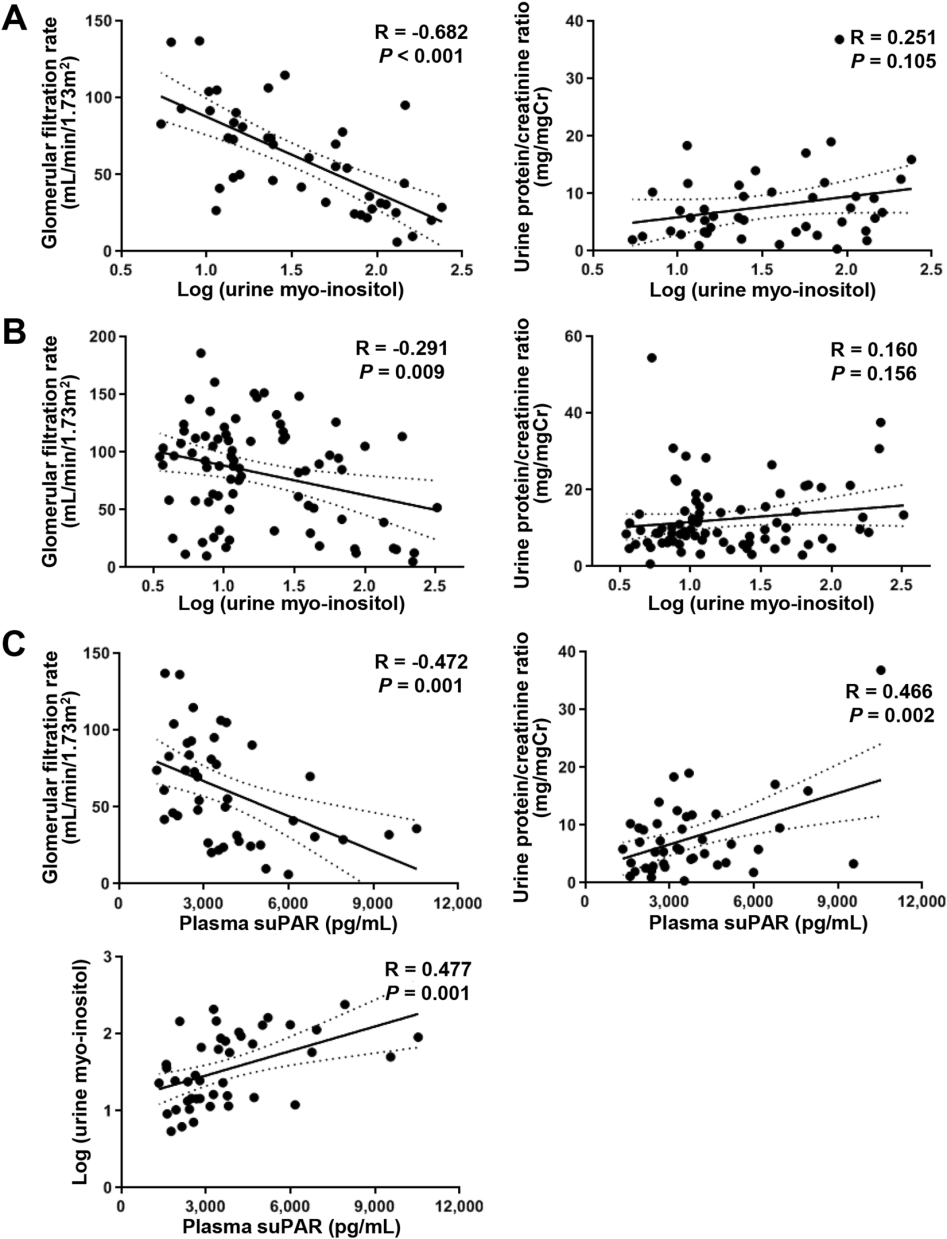
Association between urinary myo-inositol and clinical parameters. (**A**) In the FSGS patients. Log-transformed urinary myo-inositol significantly increased as eGFR decreased (R = −0.682, P < 0.001). Although the association with uPCr was not statistically significant, there was a trend for a positive relationship between uPCr and log-transformed urinary myo-inositol (R = 0.251, P = 0.105). (**B**) In the MCD patients. There was a weaker association between urinary myo-inositol and eGFR than that in the FSGS patients (R = −0.291, P = 0.009). The relationship with uPCr was not statistically significant (R = 0.160, P = 0.156). (**C**) Association between suPAR and clinical parameters in FSGS patients. The plasma concentration of suPAR showed a negative relationship with eGFR and a positive relationship with uPCr (R = −0.472, P = 0.001; R = 0.466, P = 0.002). The plasma concentration of suPAR was significantly associated with log-transformed urinary myo-inositol in FSGS patients (R = 0.477, P = 0.001).

We analyzed the association of urinary myo-inositol with baseline clinical parameters at the time of kidney biopsy. In the FSGS patients, as estimated glomerular filtration rate (eGFR) decreased, log-transformed urinary myo-inositol significantly increased (Pearson correlation coefficients; R = −0.682, P < 0.001; Fig. 2A). The relationship between urinary myo-inositol and eGFR was closer and more significant in the FSGS patients than in the MCD patients (Fig. 2B). However, the relationship with spot urine protein-creatinine ratio (uPCr) was not significant in either group. Although not statistically significant, there was a trend toward a positive relationship between uPCr and log-transformed urinary myo-inositol in the FSGS patients.

Next, the linear relationship between log-transformed urinary myo-inositol and plasma concentration of suPAR was investigated in FSGS patients. The plasma concentration of suPAR showed a positive relationship with uPCr and a negative relationship with eGFR in FSGS patients (Fig. 2C). Then, we verified that the plasma concentration of suPAR was significantly related to log-transformed urinary myo-inositol (Pearson correlation coefficients; R = 0.447, P = 0.001).

### Urinary myo-inositol for discrimination of FSGS from MCD

We examined whether the addition of urinary myo-inositol could better discriminate FSGS compared to traditional clinical parameters, including proteinuria and suPAR (Table 2). In the receiver operating characteristic (ROC) analysis, the addition of urinary myo-inositol did not increase the area under the curve (AUC) for the discrimination of FSGS. However, the inclusion of urinary myo-inositol exhibited modest but significant increases in the integrated discrimination improvement (IDI) (uPCr + log(urine myo-inositol) 0.097 [95% confidence intervals (CI), 0.041–0.154], P = 0.001; uPCr + suPAR + log(urine myo-inositol) 0.122 [95% CI, 0.057–0.187]; P < 0.001). Furthermore, an analysis using the category-free net reclassification improvement (NRI) showed that the inclusion of urinary myo-inositol significantly improved the net risk reclassification by 49.1% (95% CI, 13.0–85.3%; P = 0.008) and 59.9% (95% CI, 24.3–95.5%; P = 0.028) compared to the traditional clinical parameters.

**Table 2:** Incremental value of urinary myo-inositol over traditional clinical parameters for discriminating FSGS from MCD.

	ROC analysis (DeLong test)		IDI analysis		Category-free NRI analysis	
	AUC (95% CI)	P-value	IDI (95% CI)	P-value	NRI (95% CI)	P-value
uPCr	0.702 (0.601–0.803)		reference		reference	
uPCr + suPAR	0.768 (0.681–0.855)	0.075	0.074 (0.029–1.120)	0.001	0.634 (0.284–0.984)	<0.001
uPCr + log(urine myo-inositol)	0.770 (0.683–0.858)	0.128	0.097 (0.041–0.154)	0.001	0.491 (0.130–0.853)	0.008
uPCr + suPAR + log(urine myo-inositol)	0.783 (0.700–0.865)	0.087	0.122 (0.057–0.187)	<0.001	0.599 (0.243–0.955)	0.001
uPCr + eGFR + suPAR + log(urine myo-inositol)	0.790 (0.706–0.873)	0.066	0.154 (0.084–0.224)	<0.001	0.785 (0.441–1.129)	<0.001

^*^AUC, area under the curve; eGFR, estimated glomerular filtration rate; IDI, integrated discrimination improvement; NRI, net reclassification improvement; ROC, receiver-operating characteristic; suPAR, soluble urokinase plasminogen-type activator receptor; uPCR, urine protein-creatinine ratio.

### Association between urinary Myo-inositol and clinical outcomes

**Figure 3:**
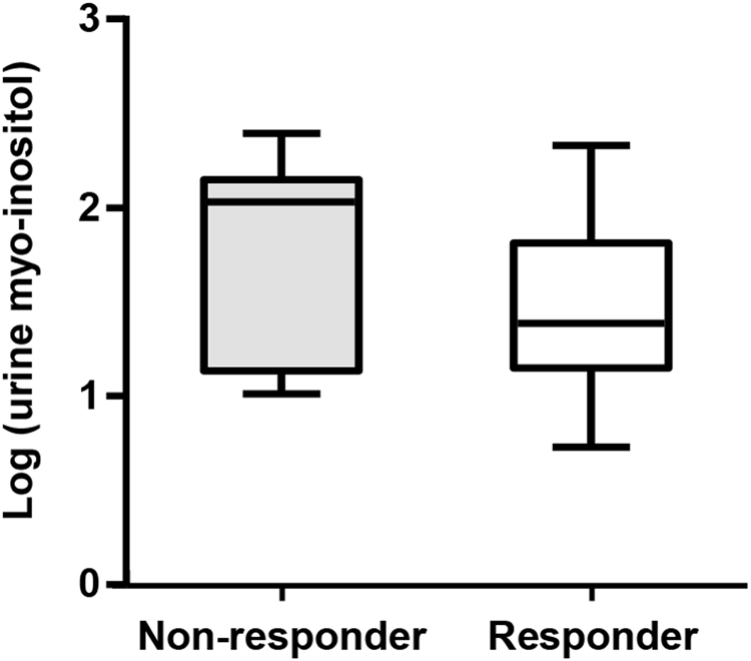
Association between urinary myo-inositol and clinical outcomes in the FSGS patients. Although the difference was not significant, the log-transformed urinary myo-inositol level was higher in the nonresponders compared with the responders (nonresponders *vs*. responders, 2.02 (1.43, 2.06) *vs*. 1.38 (1.30, 1.61); P = 0.062).

After division of the FSGS patients by their response to initial treatment, there were 30 (69.8%) responders and 13 (30.2%) nonresponders. The log-transformed urinary myo-inositol level was higher in the nonresponders than in the responders (median (interquartile range (IQR)); 2.02 (1.43, 2.06); 1.38 (1.30, 1.61)) (Fig. 3). Although the difference was statistically nonsignificant, a clear trend was shown. Binary logistic regression analysis adjusted for age, sex, comorbidities, and baseline renal function also demonstrated that as the log-transformed urinary myo-inositol level increased, the probability of responding to the initial treatment decreased (odds ratios (OR) 0.09; 95% CI, 0.01–0.75; P = 0.025) (Table 3).

**Table 3:** The association between urinary myo-inositol and response to initial treatment.

Logistic regression analysis		
Model	OR (95% CI)	*P*-value
Model 1	0.23 (0.05–1.09)	0.065
Model 2	0.09 (0.01–0.66)	0.017
Model 3	0.10 (0.01–0.75)	0.026
Model 4	0.09 (0.01–0.75)	0.025

Model 1: log(urine myo-inositol).

Model 2: log(urine myo-inositol) + sex + age.

Model 3: log(urine myo-inositol) + sex + age + hypertension + diabetes.

Model 4: log(urine myo-inositol) + sex + age + hypertension + diabetes + urine protein-creatinine ratio.

We also evaluated whether the addition of urinary myo-inositol could better predict the response to initial treatment than traditional risk factors, including sex, age, and level of proteinuria (Table 4). In the ROC analysis, the addition of urinary myo-inositol did not increase the AUC for the prediction of nonresponders. However, the inclusion of urinary myo-inositol showed significant increases in the IDI (sex + age + log(urine myo-inositol) 0.152 [95% CI, 0.018–0.287], P = 0.026; sex + age + uPCr + log(urine myo-inositol) 0.154 [95% CI, 0.019–0.290], P = 0.026; sex + age + uPCr + suPAR + log(urine myo-inositol) 0.155 [95% CI, 0.019–0.290], P = 0.025). The analysis using the category-free NRI verified that the combination of both suPAR and urinary myo-inositol significantly improved the net risk reclassification by 71.8% (95% CI, 11.3–132.3%; P = 0.020) compared to the traditional risk factors.

**Table 4:** Incremental value of urinary myo-inositol over traditional risk factors for predicting nonresponders among the FSGS patients.

	ROC analysis (DeLong test)		IDI analysis		Category-free NRI analysis	
	AUC (95% CI)	P-value	IDI (95% CI)	P-value	NRI (95% CI)	P-value
Sex + Age + uPCr	0.628 (0.447–0.809)		reference		reference	
Sex + Age + log(urine myo-inositol)	0.736 (0.566–0.906)	0.385	0.152 (0.018–0.287)	0.026	0.497 (−0.134–1.129)	0.123
Sex + Age + uPCr + log(urine myo-inositol)	0.749 (0.582–0.915)	0.329	0.154 (0.019–0.290)	0.026	0.564 (−0.063–1.192)	0.078
Sex + Age + uPCr + suPAR + log(urine myo-inositol)	0.751 (0.586–0.916)	0.317	0.155 (0.019–0.290)	0.025	0.718 (0.113–1.323)	0.020

*AUC, area under the curve; IDI, integrated discrimination improvement; NRI, net reclassification improvement; ROC, receiver-operating characteristic; suPAR, soluble urokinase plasminogen-type activator receptor; uPCR, urine protein-creatinine ratio.

### *In vitro* study

**Figure 4:**
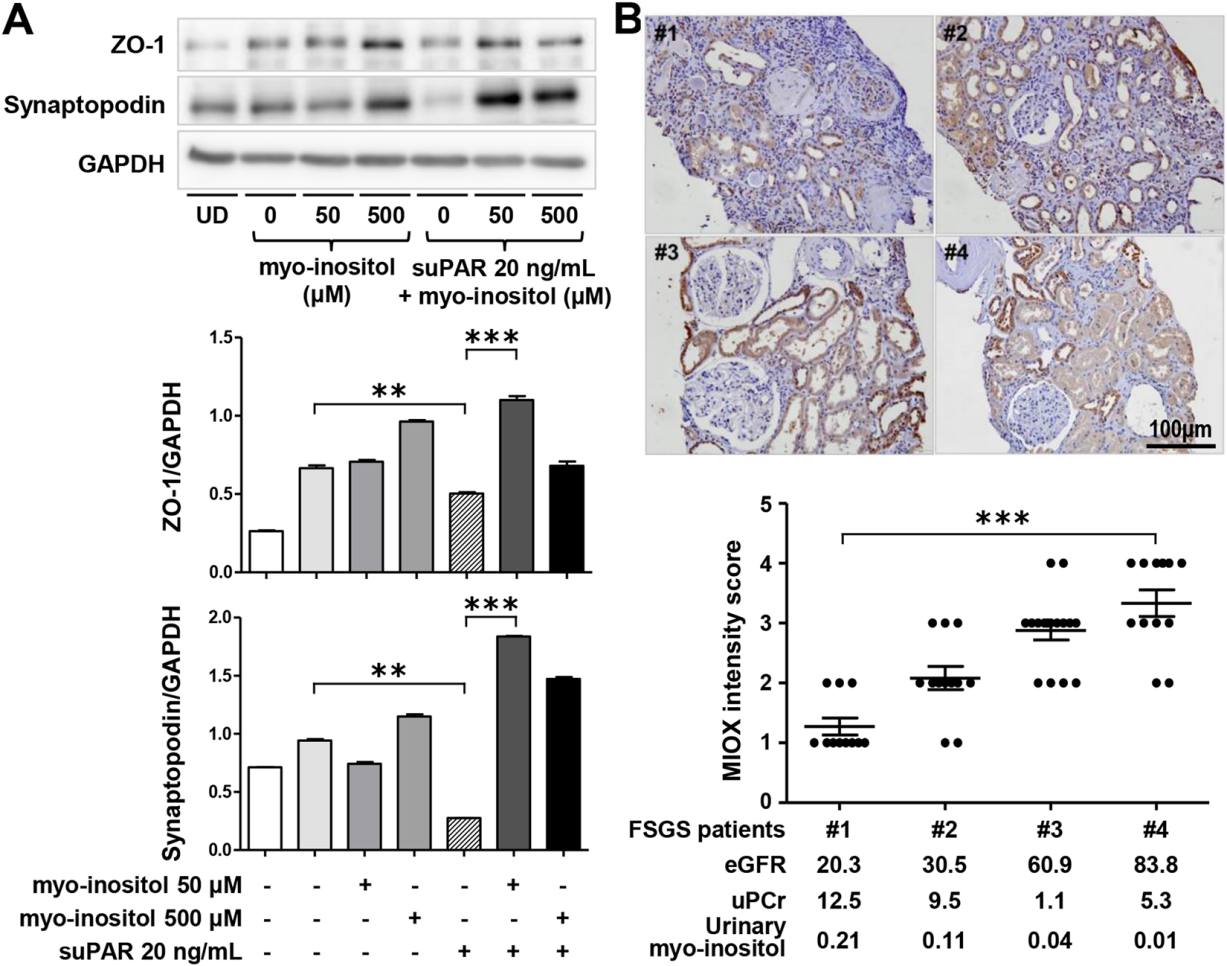
(**A**) Effect of myo-inositol treatment in the FSGS *in vitro* model. Administration of recombinant suPAR to podocytes significantly decreased synaptopodin and ZO-1 expression. However, myo-inositol treatment increased the expression of these markers again. These results represent one of three independent experiments. After densitometry analysis of the western blotting results for all cellular protein samples, the results of representative samples are shown in the figure. The data are shown as the mean ± standard deviation and were compared using Student’s t-test (*P < 0.05, **P < 0.01, ***P < 0.001). **(B**) In FSGS patients, as myo-inositol increased, myo-inositol oxygenase expression significantly decreased. The degree of myo-inositol oxygenase tissue expression was not related to uPCR but was directly proportional to eGFR (***P < 0.001; magnification: 200x).

Additionally, we examined the effect of myo-inositol treatment in the *in vitro* FSGS model. When recombinant suPAR was administered to podocytes for 24 hours, the expression of synaptopodin as an actin-associated protein, which is involved in cell shape and motility, and zonula occludens-1 (ZO-1), a tight junction protein-1, significantly decreased (Fig. 4A). On the other hand, myo-inositol treatment ameliorated the decreased expression of synaptopodin and ZO-1 (see Supplementary Fig. S5).

### Immunohistochemistry of Myo-inositol oxygenase

Next, we examined the expression of myo-inositol oxygenase (MIOX), which is primarily expressed in the kidneys and is known to be a part of the inositol catabolism pathway, in patients with FSGS and MCD. As shown in Fig. 4B, there was an inverse relationship between the urinary myo-inositol level and MIOX tissue expression level; as myo-inositol increased, MIOX expression significantly decreased. The degree of MIOX tissue expression was not related to uPCR but was directly proportional to eGFR.

## Discussion

In this study, we reported urinary metabolite profiles of FSGS patients compared to those of MCD patients or healthy controls, and we found a significant difference in urinary myo-inositol levels among these groups. Urinary myo-inositol levels were significantly related to the clinical parameters of FSGS patients measured at diagnosis and increased the ability to distinguish FSGS patients from MCD patients compared to traditional clinical parameters. In addition, we demonstrated the association between urinary myo-inositol and clinical outcomes and that the addition of urinary myo-inositol could better predict the response to initial treatment compared to traditional risk factors. Moreover, we investigated the effects of myo-inositol treatment in an *in vitro* FSGS model, and we found that MIOX increased in proportion to eGFR in FSGS human kidney tissue, while it was inversely related to urinary myo-inositol.

Various studies with metabolomics have been actively carried out in the field of glomerular diseases. Differential urinary metabolite profiles were identified between lupus nephritis and FSGS^12^, and Xianfu Gao *et al*.^13^ found that metabolites in urine and blood markedly differ with the levels of proteinuria in patients with membranous nephropathy (MN). However, these studies presented only cross-sectional analysis results limited to a small number of patients. Blood metabolites were found to be highly elevated in IgA nephropathy (IgAN) patients compared to those in healthy controls^14^; however, there was no difference according to the risk of disease in this cross-sectional analysis. In an NMR-based metabolomics study, FSGS showed a significantly different urinary metabolic profile compared to three other GNs (MN, MCD, and IgAN) and healthy controls, suggesting that this approach could be a noninvasive way to diagnose and differentiate FSGS^15^. In addition, a recent study using mass spectrometry reported the differences in urinary metabolite profiles and combined them to form an integrated metabolic signature, which allows us to distinguish three distinct nephrotic syndromes (MCD, FSGS, and MN) from each other and from the control group^16^. However, these studies included only a small number of patients in each group and did not reveal whether such metabolic profiles predict or are correlated with long-term clinical outcomes. In this study, we found early markers that differentiate FSGS using a considerable number of urine samples. In addition to the simple correlation with the various clinical parameters at the time of diagnosis, a longitudinal analysis also confirmed the association with the clinical prognosis.

Myo-inositol, identified as a meaningful urinary metabolite in this study, is produced from glucose-6-phosphate and acts as a precursor of inositol phosphates and a component of phosphatidylinositols. It is involved in cell survival and growth, the central nervous system, reproduction, metabolism and osteogenesis, and the kidney is the most important organ involved in its catabolism as well as its synthesis in humans^17^. Myo-inositol was elevated in the serum of incident ESRD and dialysis patients^18^ and was also reported as a uremic retention solute associated with progression to ESRD in type 2 diabetes patients^19^. Diémé B *et al*.^20^ found that urinary myo-inositol varied over time in kidney transplant recipients and that it was associated with short-term graft outcome, suggesting that it may be a noninvasive method of monitoring kidney transplant recipients. Recently, Gil RB *et al*. reported that inhibition of tubular transport and increased urine levels of myo-inositol, a renal osmolyte, resulted in an osmolyte imbalance and aggravated chronic kidney disease (CKD) progression with renal cell damage. Urinary myo-inositol was closely related to eGFR decrease, predicting that it could be used as a prognostic marker for CKD progression^21^. Moreover, Dutta RK *et al*.^22^ demonstrated that overexpression of MIOX promotes degradation of myo-inositol and decreases myo-inositol levels, worsening cisplatin-induced acute kidney injury. These results are consistent with our findings that urinary myo-inositol is increased in FSGS patients with reduced renal function (increased suPAR level) or reduced MIOX expression and that treatment with myo-inositol in the *in vitro* FSGS model resulted in an increase in the expression of various markers, which was similar to the attenuation of the disease. These findings suggest that myo-inositol is associated with tubular dysfunction and disorder of myo-inositol degradation or secretion rather than FSGS disease-specific pathogenesis^23^. This finding was confirmed in a historical study by Pitkänen E, reporting that the urinary appearance of myo-inositol was closely related to kidney function, and the estimation of urinary myo-inositol was useful in the evaluation of kidney function^24^. However, when the cases were divided into groups according to basal renal function, urinary myo-inositol was found to be higher in FSGS than control or MCD in both discovery and validation sets (see Supplementary Fig. S6). There was no difference in basal renal function between FSGS and MCD patients with eGFR < 60 mL/min/1.73 m^2^ in both discovery and validation sets. The patients with FSGS showed no improvement in renal function, whereas renal impairment of MCD patients was found to recover significantly after treatment. As a result of all this, the difference in urinary myo-inositol levels in FSGS and MCD patients verified to be significant regardless of basal renal function (see Supplementary Table S4).

This study has a strong point that we have performed a longitudinal analysis using a long-term clinical outcome in addition to cross-sectional analysis, and we demonstrated that myo-inositol improves the ability to differentiate FSGS patients and that it improves the predictive power of long-term clinical prognosis; therefore, myo-inositol may be an important indicator in the diagnosis and treatment of patients with FSGS. Furthermore, it is important to be able to evaluate and predict various indicators by measuring metabolites using urine specimens, which can be obtained easily and in large quantities, instead of invasive and nonrepeatable renal biopsy.

There are some limitations to our study. First, the number of patients is too low to generalize the results of the study. Second, there was no clear evaluation of the mechanism. Finally, several previous studies have found correlations of urinary myo-inositol with various diseases, especially its close association with renal function. Furthermore, we did not compare FSGS with the other disease control group (non-nephrotic CKD control group), and thus, it is difficult to suggest urinary myo-inositol is a specific biomarker for only FSGS. Nevertheless, this study showed the association with a long-term clinical outcome, and we conducted various analyses to clarify the relationship between urinary myo-inositol and therapeutic response. The sample size in this study was also appropriate in terms of marker discovery.

In conclusion, urinary myo-inositol differentiates FSGS patients from MCD patients and correlates with various clinical parameters of FSGS patients. In addition, this metabolite correlates with the clinical outcome of patients with FSGS and improved prediction of the treatment response. The relationship between myo-inositol and decreased renal function or MIOX expression suggests a link between myo-inositol and tubular dysfunction. As myo-inositol treatment alleviates the severity of the FSGS *in vitro* model, further studies are needed to confirm the FSGS-specific association of myo-inositol. In the future, it is necessary to confirm the findings of this study through comparative studies with other disease groups.

## Methods

### Study design and population

From December 2010 to March 2017, patients who were diagnosed with MCD or FSGS through kidney biopsy were selected from the kidney disease cohort studies conducted at Seoul National University Hospital and Seoul National University Boramae Medical Center (see Supplementary Fig. S7). After the exclusion of secondary causes based on the results of biopsy and the judgment of nephrologists, 80 MCD patients and 43 FSGS patients were included in the discovery set of this study. Urine samples collected at the time of kidney biopsy were used in the analysis, and all patients who started treatment prior to biopsy were excluded. We also enrolled a control population of 61 patients.

As a validation set, we used a total of 61 urine samples (see Supplementary Fig. S7). This study was approved by the Institutional Review Board of Seoul National University Boramae Medical Center and Seoul National University Hospital (No. 16-2016-58/061, 1610-001-794), and the patients provided informed consent prior to recruitment into each cohort. All clinical investigations were conducted in accordance with the guidelines of the 2013 Declaration of Helsinki; see Supplementary Methods.

### Clinical data collection

Data regarding clinical parameters, including age, sex, body mass index, blood pressure, smoking history, comorbidities, and renal function (serum creatinine (sCr) and spot uPCr), were collected at the time of kidney biopsy. Serum Cr, eGFR, and uPCr measured at the last visit were also collected; see Supplementary Methods.

Responses were classified into the following two groups: responders, patients who had CR or partial remission defined according to KDIGO guidelines; and nonresponders, patients who had never undergone CR or partial remission during the treatment period.

### Sample preparation for metabolite analysis

Urine samples were filtered through Amicon^®^ Ultra centrifugal filters for 500 μL–3 K (Millipore, Billerica, MA, USA) at 12,000 rpm for 10 minutes at 4 °C to remove protein. The resulting 300-μL supernatant from the urine sample was mixed with 300 μL of 0.2 M sodium phosphate buffer (pH 7.0) and 1 mM sodium azide in deuterium oxide (D_2_O). After adjusting the pH to 7.0 ± 0.1, 540 μL of sample was mixed with 60 μL of 5 mM 3-(trimethylsilyl) propionic 2,2,3,3-acid (TSP) in D_2_O; see Supplementary Methods.

### ^1^H NMR experiment

One-dimensional (1D) ^1^H NMR spectra were acquired with an Ascend 800-MHz AVANCE III HD Bruker spectrometer (Bruker BioSpin AG). The processed NMR spectra were imported into Chenomx for identification and quantification, and the 800-MHz Chenomx library (ver. 7.1, Chenomx, Edmonton, AB, Canada) was used to identify individual compounds. The urinary concentrations were normalized to the levels of creatinine (metabolite μM/creatinine mM); see Supplementary Methods.

### Measurement of plasma soluble urokinase plasminogen activator receptor

A human uPAR Quantikine ELISA Kit (R&D Systems, Minneapolis, MN, USA) was used to measure suPAR concentrations in plasma samples obtained at the time of kidney biopsy; see Supplementary Methods.

### *In vitro* study

For the *in vitro* model mimicking FSGS, an immortalized human podocyte cell line, which was a gift from Dr. Peter Mundel, was used as described in our previous study^25,26^. After podocyte characterization and differentiation were confirmed, the podocytes were serum starved for 24 hours and treated with human recombinant suPAR (20 ng/mL; R&D Systems) and myo-inositol (50 and 500 μM; Sigma-Aldrich, St. Louis, MO, USA).

After 24 hours, the cells were collected, and protein was extracted. Primary antibodies targeting glyceraldehyde 3-phosphate dehydrogenase (GAPDH, Cell Signaling, Danvers, MA, USA), synaptopodin (Progen, Heidelberg, Germany), and ZO-1 (Cell Signaling) were used. Anti-rabbit IgG (Cell Signaling) was used as a secondary antibody. Labeled proteins were detected using an enhanced chemiluminescence system (LAS-4000; Fujifilm, Tokyo, Japan). The target molecule expression levels were normalized with respect to GAPDH expression. Densitometry was performed using the gel analysis function of ImageJ software (National Institutes of Health, Bethesda, Maryland, USA); see Supplementary Methods.

### Immunohistochemistry of biopsy slides

Unstained slides from the study population were used. Staining was performed using polyclonal MIOX antibody (1:1000; Thermo Fisher Scientific, Waltham, MA, USA) at 4 °C overnight, followed by incubation with dextran polymer conjugated with horseradish peroxidase (GBI Labs, Bothell, WA, USA) for 5 minutes at room temperature. Finally, all sections were counterstained with Mayer’s hematoxylin (ScyTek Laboratories, West Logan, UT, USA) and evaluated under light microscopy. The MIOX score was graded in a blinded fashion by a kidney pathologist. The score was expressed semiquantitatively from 1 to 4 as follows: score 1, no staining or faint staining in a few tubules; score 2, mild staining; and scores 3 and 4, moderate and strong staining, respectively; see Supplementary Methods.

### Statistical analysis

Categorical variables described as frequencies and proportions were compared using chi-squared tests. The nonnormally distributed variables were expressed as medians with IQR and were compared using the Mann-Whitney U or Kruskal-Wallis test. Pearson correlation coefficients were determined to explore the linear relationship between log-transformed urinary metabolites and various clinical parameters including eGFR, uPCr, and plasma concentration of suPAR.

All NMR results are expressed as the median fold change. One-way analysis of covariance (ANCOVA) after conversion into ranked variables was used to detect differences among FSGS, MCD, and healthy control groups, while age, sex, diabetes, and hypertension status were adjusted as potential confounders. The P-values from ANCOVAs were adjusted using the Bonferroni correction for multiple comparisons. Using statistical analysis system (SAS university edition, Cary, NC, USA), propensity scores were used to match age, sex, and diabetes status (n = 43 in each group). PCA and PLS-DA were conducted using SIMCA-P+ (ver. 12.0, Umetrics, Umea, Sweden); see Supplementary Methods.

The contribution of urinary myo-inositol for discriminating FSGS patients from MCD patients or identifying the FSGS patients at high risk of being nonresponder was examined by the area under the receiver operating characteristic curve (AUROC). The discrimination performances were assessed with the DeLong test, NRI, and IDI. A simple logistic regression model was used to calculate unadjusted ORs and 95% CIs for responses to initial treatment. A P*-*value < 0.05 was considered significant. Statistical analyses were performed with SPSS software, version 20.0 K (SPSS Inc., Chicago, IL, USA) and R version 3.5.0 (http://www.r-project.org).

## Supplementary information

Supplementary Information

## Data Availability

The datasets generated during and/or analyzed during the current study are available from the corresponding authors on reasonable request.
