# Supplementary material for: Urinary myo-inositol is associated with the clinical outcome in focal segmental glomerulosclerosis
Source: Sci Rep. 2019 Oct 11;9:14707. doi: 10.1038/s41598-019-51276-9 (PMC6789025; doi:10.1038/s41598-019-51276-9)

Urinary myo-inositol is associated with the clinical outcome in focal segmental glomerulosclerosis

Jung Nam An[1*], Jin Seong Hyeon[2,3*], Youngae Jung[2], Young Wook Choi[1], Jin Hyuk Kim[1], Seung Hee Yang[4], Sohee Oh[5], Soie Kwon[6], Sang-Ho Lee[7], Jang-Hee Cho[8], Sun-Hee Park[8], Hunjoo Ha[3], Dong Ki Kim[6,9], Jung Pyo Lee[1,9*] and Geum-Sook Hwang[2,10*]

Supplementary Information

Supplementary Methods

Table S1. Urinary metabolites showing significant differences among the focal segmental glomerulosclerosis, minimal change disease, and healthy control groups

Table S2. Significantly different urinary metabolites among the FSGS and MCD patients and the healthy control group, after matching for age, sex, and diabetes status

Table S3. Demographics and baseline characteristics of the validation set

Table S4. Changes in eGFR before and after treatment in FSGS or MCD patients below eGFR 60 mL/min/1.73 m^2

Figure S1. 1D ^1H NMR spectra of urine samples from healthy controls (A), FSGS patients (B), and MCD patients (C)

Figure S2. Representative 2D ^1H-^1H COSY NMR spectrum of urine samples from FSGS patients

Figure S3. Representative 2D ^1H-^{13}C HSQC NMR spectrum of urine samples from FSGS patients

Figure S4. Multivariate statistical analyses of urine samples from the validation set

Figure S5. Full-length gels and blots, and multiple exposures of GAPDH (A), Synaptopodin (B), and ZO-1 (C)

Figure S6. Subgroup analysis according to basal renal function

Figure S7. Enrollment of the discovery set and the validation set

Supplementary Methods

Study Design and Population

From December 2010 to March 2017, patients who were diagnosed with MCD or FSGS through kidney biopsy were selected from the kidney disease cohort studies conducted at Seoul National University Hospital and Seoul National University Boramae Medical Center (**see Supplementary Fig. S7**). These prospective cohort studies have biobanks for blood, urine, genomic DNA, and biopsied tissues of patients who underwent kidney biopsy and provided informed consent. After the exclusion of secondary causes based on the results of biopsy and the judgment of nephrologists, 80 MCD patients and 43 FSGS patients were included in the discovery set of this study. Urine samples collected at the time of kidney biopsy were used in the analysis, and all patients who started treatment prior to biopsy were excluded. We also enrolled a control population of 61 patients who had normal renal function without recent kidney function changes and urinary abnormalities and collected residual urine samples after health examination.

As a validation set, we used a total of 61 urine samples. Twenty-six primary MCD patients and 23 primary FSGS patients were recruited from Gangdong Kyung Hee University Hospital and Gyeongbuk National University Hospital; 12 healthy control samples were extracted from Seoul National University Boramae Medical Center Human Biobank (**see Supplementary Fig. S7**).

This study was approved by the Institutional Review Board of Seoul National University Boramae Medical Center and Seoul National University Hospital (No. 16-2016-58/061, 1610-001-794), and the patients provided informed consent prior to recruitment into each cohort. All clinical investigations were conducted in accordance with the guidelines of the 2013 Declaration of Helsinki.

Clinical Data Collection

Data regarding clinical parameters, including age, sex, body mass index, blood pressure, smoking history, comorbidities (hypertension, diabetes, cardiovascular and cerebrovascular disease, and malignancy), and renal function (serum creatinine (sCr) and spot urine protein-creatinine ratio (uPCr)) were collected at the time of kidney biopsy. The estimated glomerular filtration rate (eGFR) was calculated using the isotope dilution mass spectrometry (IDMS) – traceable modified Modification of Diet in Renal Disease (MDRD) equation. Serum Cr, eGFR, and uPCr measured at the last visit were also collected.

Responses were classified into the following two groups: responders, patients who had CR or partial remission defined according to KDIGO guidelines; and nonresponders, patients who had never undergone complete remission (CR) or partial remission during the treatment period.

Sample Preparation for Metabolite Analysis

Prior to the nuclear magnetic resonance (NMR) experiment, the frozen urine samples were thawed at room temperature. Urine samples were filtered through Amicon® Ultra centrifugal filters for 500 μL – 3K (Millipore, Billerica, MA, USA) at 12,000 rpm for 10 minutes at 4℃ to remove protein. The resulting 300-μL supernatant from the urine sample was mixed with 300 μL of 0.2 M sodium phosphate buffer (pH 7.0) and 1 mM sodium azide in deuterium oxide (D_2O). After adjusting the pH to 7.0 ± 0.1, 540 μL of sample was mixed with 60 μL of 5 mM 3-(trimethylsilyl) propionic 2,2,3,3-acid (TSP) in D_2O, and the 600-μL samples were placed in 5-mm Bruker SampleJet NMR tubes (Z112273, Bruker BioSpin AG, Fällanden, Switzerland).

^{1}H NMR Experiment

One-dimensional (1D) ^{1}H NMR spectra were acquired with an Ascend 800-MHz AVANCE III HD Bruker spectrometer (Bruker BioSpin AG) using a triple-resonance 5-mm CPTIC cryogenic probe. To acquire 1D ^{1}H spectra of the urine samples, Bruker standard 1D nuclear Overhauser enhancement spectroscopy (NOESY)-presat (noesypr1d) pulse sequences were used as follows: relaxation delay (*RD*) – 90° – short delay – 90° – mixing – 90°– *Acq*, with *RD* = 4.0 s, short delay = 11.3 us, *n* = 128, dummy scans = 4, acquisition time (*Acq*) = 2.0 s, and mixing time (mixing) = 10 ms. The water signal was suppressed at the water peak during the *RD* and mixing time. Fourier domain points were acquired at 65,536 data points with a spectral width of 20 ppm.

The NMR data were processed using TopSpin (ver. 3.1, Bruker BioSpin, Rheinstetten, Germany). All spectra were baseline-corrected and phase-corrected manually. The processed NMR spectra were imported into Chenomx for identification and quantification, and the 800-MHz Chenomx library (ver. 7.1, Chenomx, Edmonton, AB, Canada) was used to identify individual compounds. The assignment of ambiguous peaks due to peak overlap was confirmed by spiking with standard compounds. Signal assignment for representative samples was facilitated by the acquisition of two-dimensional (2D) correlation spectroscopy (COSY) and heteronuclear single quantum correlation (HSQC). The quantification of urinary metabolites was achieved using Chenomx, which used the concentration of TSP to determine the concentration of individual compounds. The urinary concentrations were normalized to the levels of creatinine (metabolite μM/creatinine mM).

Measurement of Plasma Soluble Urokinase Plasminogen Activator Receptor

A human uPAR Quantikine ELISA Kit (R&D Systems, Minneapolis, MN, USA) was used to measure the concentrations of soluble urokinase plasminogen activator receptor (suPAR) in

plasma samples obtained at the time of kidney biopsy. According to the manufacturer's instructions, the frozen plasma samples were left at room temperature for 30 minutes to equilibrate and were then subjected to 5-fold dilution and duplicate testing. The absorbance was detected with an ELISA reader at 450 nm.

In Vitro Study

For the *in vitro* model mimicking FSGS, an immortalized human podocyte cell line, which was a gift from Dr. Peter Mundel, was used as described in our previous study.[12,13] Briefly, podocytes were propagated, seeded, and grown in RPMI 1640 medium (Welgene, Korea) with 10% FBS (GenDEPOT, Katy, TX, USA), 1X insulin-transferrin-selenium (Gibco, Grand Island, NY, USA), 2 mM glutamine (Biowest, Riverside, MO, USA), and 1X penicillin/streptomycin (Gibco) on fibronectin-coated culture plates at 33℃. When the cells reached ~80% confluence, they were transferred to incubate at 37℃ for 7 days. After podocyte characterization and differentiation were confirmed, the podocytes were serum starved for 24 hours and then treated with human recombinant suPAR (20 ng/mL; R&D Systems) and myo-inositol (50 and 500 μM; Sigma-Aldrich, St. Louis, MO, USA).

After 24 hours, the cells were collected, and protein was extracted. Equal amounts (10 μg) of protein were separated on 4-10% sodium dodecyl sulfate-polyacrylamide gels and transferred onto Immobilon-P 0.45-μm polyvinylidene difluoride membranes (Millipore, Bedford, MA, USA). Primary antibodies targeting GAPDH (Cell Signaling, Danvers, MA, USA), synaptopodin (Progen, Heidelberg, Germany), and ZO-1 (Cell Signaling) were used. Anti-rabbit IgG (Cell Signaling) was used as a secondary antibody. Labeled proteins were detected using an enhanced chemiluminescence system (LAS-4000; Fujifilm, Tokyo, Japan). The target molecule expression levels were normalized with respect to GAPDH expression. Densitometry was performed using the gel analysis function of ImageJ software (National

Institutes of Health, Bethesda, MD, USA).

Immunohistochemistry of Biopsy Slides

Unstained slides from the study population were used. Paraffin-embedded kidney tissues (4-μm thick) were deparaffinized and hydrated using xylene and ethanol. Endogenous streptavidin activity was blocked using 3% hydrogen peroxide. Then, staining was performed using polyclonal myo-inositol oxygenase (MIOX, 1:1000; Thermo Fisher Scientific, Waltham, MA, USA) at 4℃ overnight, followed by incubation with dextran polymer conjugated with horseradish peroxidase (GBI Labs, Bothell, WA, USA) for 5 minutes at room temperature. Finally, all sections were counterstained with Mayer's hematoxylin (ScyTek Laboratories, West Logan, UT, USA) and evaluated under light microscopy. The MIOX score was graded in a blinded fashion by a kidney pathologist. The score was expressed semiquantitatively from 1 to 4 as follows: score 1, no staining or faint staining in a few tubules; score 2, mild staining; and scores 3 and 4, moderate and strong staining, respectively.

Statistical Analysis

Categorical variables described as frequencies and proportions were compared using chi-squared tests. After a test for normality, the nonnormally distributed variables were expressed as medians with interquartile range (IQR) and were compared using the Mann-Whitney U or Kruskal-Wallis test. Pearson correlation coefficients were determined to explore the linear relationship between log-transformed urinary metabolites and various clinical parameters including eGFR, uPCr, and plasma concentration of suPAR.

All NMR results are expressed as the median fold change. One-way analysis of covariance (ANCOVA) after conversion into ranked variables was used to detect differences among FSGS, MCD, and healthy control groups, while age, sex, diabetes, and hypertension status

were adjusted as potential confounders. The *P*-values from ANCOVAs were adjusted using the Bonferroni correction for multiple comparisons. Using statistical analysis system (SAS university edition, Cary, NC, USA), propensity scores were used to match age, sex, and diabetes status (n=43 in each group). The resultant NMR data were imported into SIMCA-P+ (ver. 12.0, Umetrics, Umea, Sweden) for chemometric analysis. Principal component analysis (PCA) is a representative unsupervised method used to analyze samples without information from groups and was performed to examine the intrinsic variation in the data set. The models were described by R^2 and Q^2 parameters. R^2 is defined as explained variation by the models and indicates the goodness of fit. Q^2 is defined as predicted variation by the models and indicates predictability. Partial least squares discriminant analysis (PLS-DA) was used to maximize class discrimination and identify a characteristic biomarker. Score plots and loading plots were obtained from the PLS-DA model.

The contribution of urinary myo-inositol for discriminating FSGS patients from MCD patients or identifying the FSGS patients at high risk of being a nonresponder was examined by the area under the receiver operating characteristic curve (AUROC). The discrimination performances were assessed with the DeLong test, net reclassification improvement (NRI), and integrated discrimination improvement (IDI). A simple logistic regression model was used to calculate unadjusted odds ratios (ORs) and 95% confidence intervals (CIs) for responses to initial treatment. A P-value < 0.05 was considered significant. Statistical analyses were performed with SPSS software, version 20.0 K (SPSS Inc., Chicago, IL, USA) and R version 3.5.0 (http://www.r-project.org).

Table S1. Urinary metabolites showing significant differences among the focal segmental glomerulosclerosis, minimal change disease, and healthy control groups

Pathway	Metabolite	Control *vs.* FSGS	Control *vs.* MCD	FSGS *vs.* MCD
Glycolysis/ glycogenesis	Glucose	5.43***	1.57***	3.46*
	Lactate	1.80**	1.03	1.74
Galactose	Myo-inositol	2.34***	0.94	2.48**
	Sucrose	4.34**	3.48***	1.25
Fructose and mannose	Mannitol	4.85***	3.09***	1.57
TCA cycle	Citrate	0.50***	0.49***	1.01
	cis-Aconitate	0.52***	0.63***	0.82
	trans-Aconitate	1.39***	1.27***	1.09
	Fumarate	2.14**	1.50**	1.43
Tyrosine	Alanine	1.80***	1.30*	1.38
	Tyrosine	1.33*	1.29*	1.04
Creatine	Guanidoacetate	0.34***	0.37***	0.93
Glutathione	Pyroglutamate	1.20*	1.15**	1.05
Glycerol	Glycerol	0.08***	0.07***	1.14
Glycine	Threonine	1.74**	1.43**	1.22
BCAA	3-Hydroxyisovalerate	0.55***	0.48***	1.14
	Isoleucine	1.69***	1.78***	0.95
	Leucine	1.85***	2.04***	0.91
	Valine	1.49***	1.57***	0.95
Choline	Betaine	2.38***	1.71**	1.39
	Choline	3.30***	2.29***	1.44
	Ethanolamine	0.68***	0.68***	0.99
Microbial	3-Indoxylsulfate	0.55**	0.53*	1.04
	Isobutyrate	0.66*	0.71***	0.93
	Methylamine	0.64*	0.77	0.83
Histidine	Histidine	0.64**	0.40***	1.62
Purine	1-Methylnicotinamide	0.56***	0.73*	0.77
	Hypoxanthine	0.30***	0.40***	0.75
Pyrimidine	Uracil	0.36***	0.51***	0.71

All models were adjusted for age, sex, diabetes, and hypertension status.

All results are expressed as the median fold change and were evaluated by one-way analysis of covariance (ANCOVA) with Bonferroni's multiple comparisons test ($P < 0.05$).

*P < 0.05, **P < 0.01, ***P < 0.001 between the indicated groups.

Table S2. Significantly different urinary metabolites among the FSGS and MCD patients and the healthy control group, after matching for age, sex, and diabetes status

Pathway	Metabolite	Control vs. FSGS	Control vs. MCD	FSGS vs. MCD
Glycolysis/ glycogenesis	Glucose	5.57***	1.61***	3.46*
	Lactate	1.92**	1.06	1.81
Galactose	myo-Inositol	2.35**	0.99	2.38*
	Sucrose	3.99**	3.30**	1.21
Fructose and mannose	Mannitol	3.94***	2.97***	1.33
TCA cycle	Citrate	0.51***	0.52**	0.98
	Fumarate	2.45**	1.82**	1.35
	cis-Aconitate	0.53***	0.65***	0.82
	trans-Aconitate	1.35***	1.25***	1.08
Tyrosine	Alanine	1.84*	1.48	1.24
	Tyrosine	1.39	1.44*	0.96
Creatine	Guanidoacetate	0.35***	0.28***	1.22
Glutathione	Pyroglutamate	1.20**	1.16**	1.04
Glycerol	Glycerol	0.08***	0.07***	1.14
Glycine	Threonine	1.90**	1.68**	1.13
BCAA	3-Hydroxyisovalerate	0.55***	0.48***	1.13
	Isoleucine	1.70***	1.84***	0.92
	Leucine	1.89***	2.14***	0.88
	Valine	1.69***	1.81***	0.93
Choline	Betaine	2.38**	1.56**	1.52
	Choline	3.53***	2.38***	1.49
	Ethanolamine	0.69***	0.68***	1.01
Microbial	3-Indoxylsulfate	0.60	0.56	1.07
	Isobutyrate	0.66*	0.72*	0.91
	Methylamine	0.62*	0.77	0.81
Histidine	Histidine	0.71**	0.45***	1.60
Purine	1-Methylnicotinamide	0.57**	0.74*	0.77
	Hypoxanthine	0.28***	0.37***	0.76
Pyrimidine	Uracil	0.37***	0.51***	0.72

All models were adjusted for age, sex, diabetes, and hypertension status.

All results are expressed as the median fold change and were evaluated by one-way analysis of covariance (ANCOVA) with Bonferroni's multiple comparisons test ($P < 0.05$).

*$P < 0.05$, **$P < 0.01$, ***$P < 0.001$ between the indicated groups.

Table S3. Demographics and baseline characteristics of the validation set

	Control (n = 12)	MCD (n = 26)	FSGS (n = 23)	P-value
**Male sex**	9 (75.0)	13 (50.0)	13 (56.5)	0.348
**Age (year)**	20 (19, 25)	52 (38, 61)	48 (28, 69)	<0.001
**Blood urea nitrogen (mg/dL)**	11.5 (10.3, 14.0)	19.5 (13.8, 27.0)	17.0 (14.0, 29.0)	0.002
**Serum creatinine (mg/dL)**	0.81 (0.70, 0.89)	0.94 (0.71, 1.44)	1.16 (0.86, 1.66)	0.014
**Estimated glomerular filtration rate (mL/min/1.73 m^2)**	113.0 (104.6, 124.0)	81.8 (50.4, 95.4)	60.9 (32.7, 92.4)	<0.001
**Urine protein/creatinine ratio (mg/mgCr)**	0.06 (0.04, 0.26)	7.74 (5.92, 12.43)	3.81 (1.50, 7.77)	<0.001
**Hypertension**	2 (16.7)	3 (11.5)	2 (8.7)	0.494
**Diabetes mellitus**	0 (0.0)	7 (26.9)	11 (47.8)	0.012

The data are presented as the median (25th, 75th percentiles) or as a number (percentage, %).

Table S4. Changes in eGFR before and after treatment in FSGS or MCD patients below eGFR 60 mL/min/1.73 m^2

Discovery set	Initial (before treatment)		
	FSGS (n=22)	MCD (n=26)	P-value
eGFR (mL/min/1.73 m^2)	31.0 (24.2, 44.8)	25.6 (16.0, 50.6)	0.438
	Follow-up (after treatment)		
	FSGS (n=22)	MCD (n=26)	P-value
eGFR (mL/min/1.73 m^2)	27.6 (16.4, 49.0)	53.7 (36.0, 72.3)	<0.001
P-value	0.681	<0.001	

Validation set	Initial (before treatment)		
	FSGS (n=11)	MCD (n=8)	P-value
eGFR (mL/min/1.73 m^2)	32.7 (28.0, 53.2)	47.5 (39.6, 50.4)	0.442
	Follow-up (after treatment)		
	FSGS (n=11)	MCD (n=8)	P-value
eGFR (mL/min/1.73 m^2)	42.0 (35.3, 49.2)	75.0 (64.4, 103.0)	0.008
P-value	0.370	<0.001	

Figure S1. 1D ^{1}H NMR spectra of urine samples from healthy controls (A), FSGS patients (B), and MCD patients (C). 1, Acetate; 2, Acetoacetate; 3, Acetone; 4, O-Acetylcarnitine; 5, cis-Aconitate; 6, trans-Aconitate; 7, Adenosine; 8, Adenosine diphosphate; 9, Alanine; 10, 2-Aminobutyrate; 11, 3-Aminoisobutyrate; 12, Arabinose; 13, Ascorbate; 14, Betaine; 15, Carnitine; 16, Choline; 17, Citrate; 18, Creatine; 19, Creatinine; 20, Dimethylamine; 21, Dimethyl sulfone; 22, Ethanolamine; 23, Formate; 24, Fumarate; 25, Gluconate; 26, Glucose; 27, Glycerol; 28, Glycine; 29, Guanidoacetate; 30, Hippurate; 31, Histidine; 32, Homovanillate; 33, 3-Hydroxybutyrate; 34, 2-Hydroxyisobutyrate; 35, 3-Hydroxyisovalerate; 36, 3-Hydroxy-3-methylglutarate; 37, 3-Hydroxyphenylacetate; 38, 4-Hydroxyphenylacetate; 39, Hypoxanthine; 40, 3-Indoxylsulfate; 41, Isobutyrate; 42, Isoleucine; 43, Lactate; 44, Leucine; 45, Maltose; 46, Mannitol; 47, Methylamine; 48, 2-Methylglutarate; 49, π-Methylhistidine; 50, τ-Methylhistidine; 51, 1-Methylnicotinamide; 52, 2-Oxoglutarate; 53, N-Phenylacetylglycine; 54, Phenylalanine; 55, Pyroglutamate; 56, Quinolinate; 57, Succinate; 58, Sucrose; 59, Taurine; 60, Threonine; 61, Trigonelline; 62, Trimethylamine N-oxide; 63, Tryptophan; 64, Tyrosine; 65, Uracil; 66, Urea; 67, Valine; 68, Xanthosine; 69, Xylose; 70, myo-Inositol.

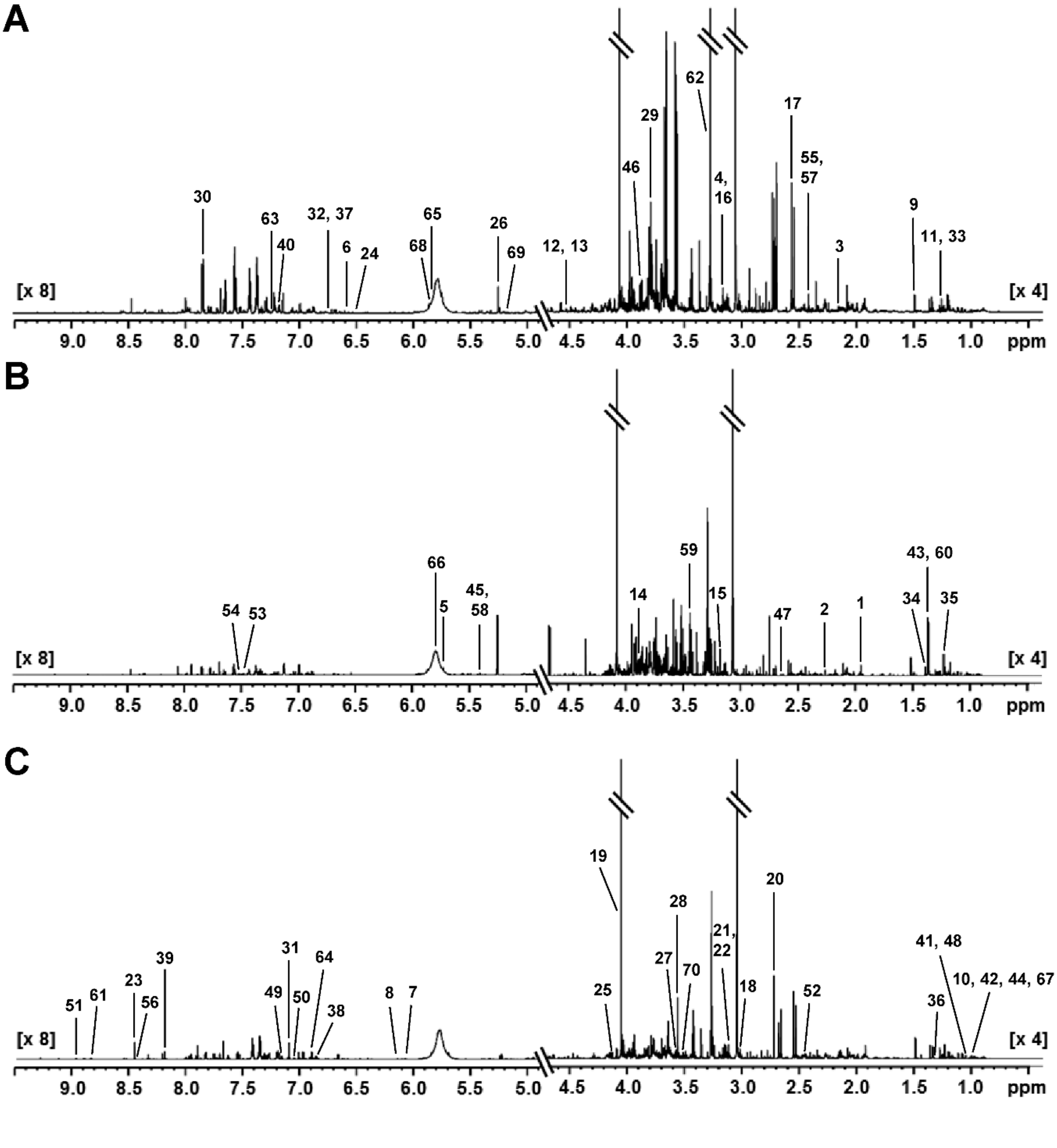

Figure S2. Representative 2D ^1H-^1H COSY NMR spectrum of urine samples from FSGS patients. 4, O-Acetylcarnitine; 10, 2-Aminobutyrate; 11, 3-Aminoisobutyrate; 41, Isobutyrate; 43, Lactate; 45, Maltose; 51, 1-Methylnicotinamide; 55, Pyroglutamate; 61, Trigonelline; 63, Tryptophan; 65, Uracil; 67, Valine; 68, Xanthosine; 69, Xylose.

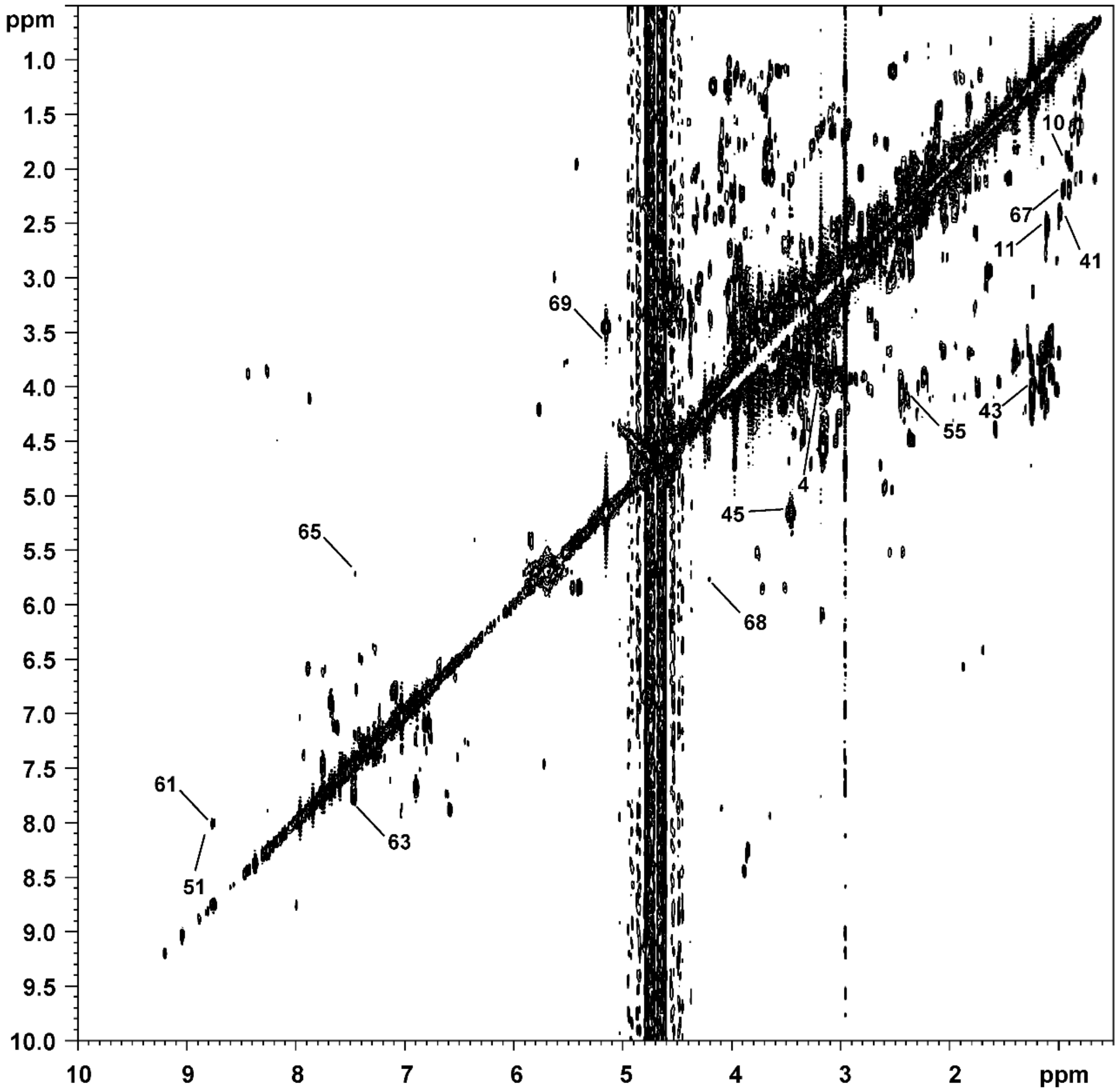

Figure S3. Representative 2D ^1H-^{13}C HSQC NMR spectrum of urine samples from FSGS patients. 4, O-Acetylcarnitine; 5, cis-Aconitate; 6, trans-Aconitate; 9, Alanine; 12, Arabinose; 14, Betaine; 15, Carnitine; 17, Citrate; 18, Creatine; 19, Creatinine; 20, Dimethylamine; 22, Ethanolamine; 25, Gluconate; 26, Glucose; 27, Glycerol; 28, Glycine; 29, Guanidoacetate; 30, Hippurate; 31, Histidine; 32, Homovanillate; 33, 3-Hydroxybutyrate; 36, 3-Hydroxy-3-methylglutarate; 37, 3-Hydroxyphenylacetate; 38, 4-Hydroxyphenylacetate; 39, Hypoxanthine; 40, 3-Indoxylsulfate; 46, Mannitol; 52, 2-Oxoglutarate; 53, N-Phenylacetylglycine; 54, Phenylalanine; 57, Succinate; 58, Sucrose; 59, Taurine; 62, Trimethylamine N-oxide; 64, Tyrosine; 70, myo-Inositol.

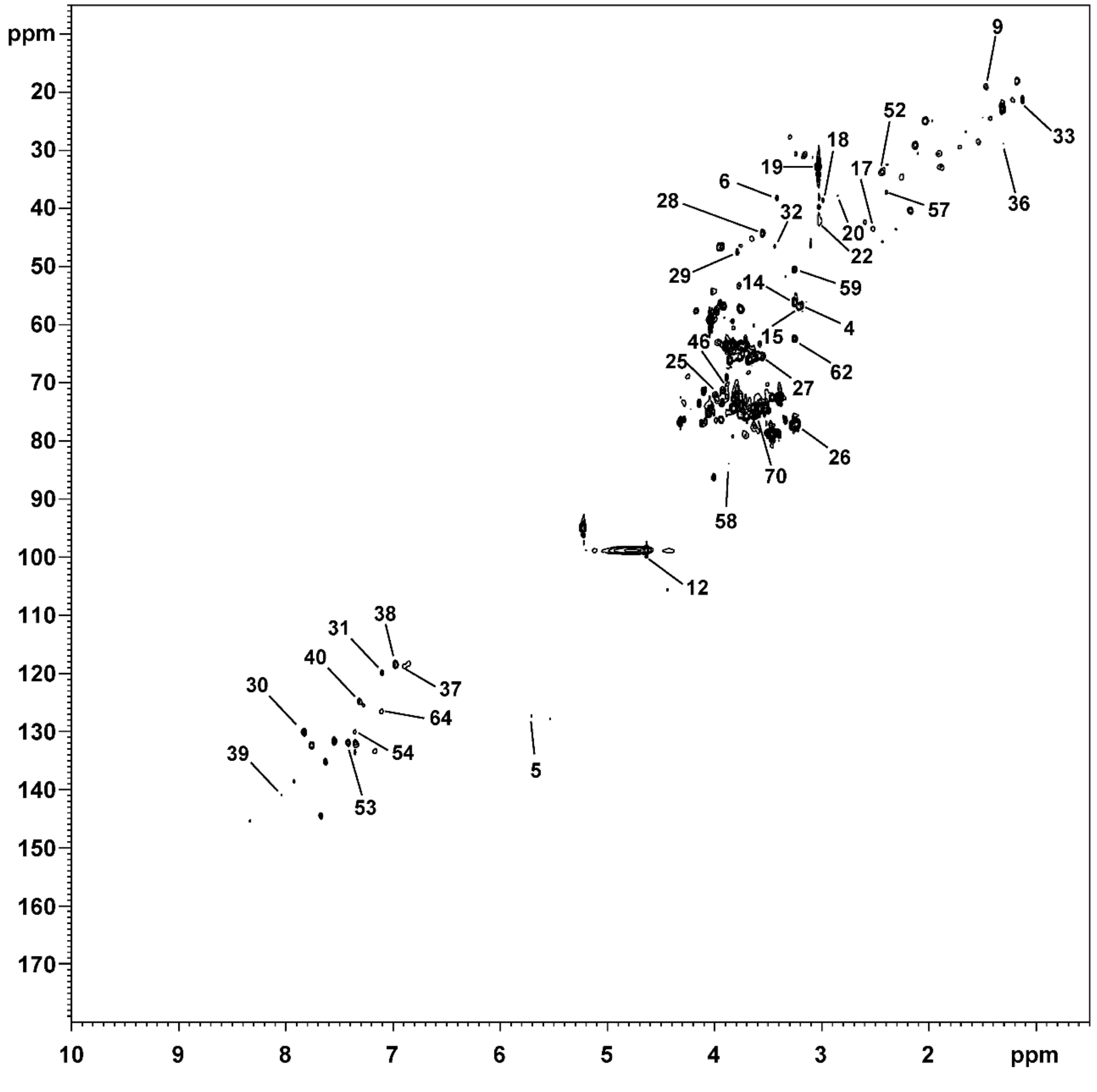

Figure S4. Multivariate statistical analyses of urine samples from the validation set. PCA (A) and PLS-DA (B) score plot obtained from ^{1}H NMR spectra of urine from FSGS, MCD, and healthy controls. Myo-inositol, which exhibited significant differences in the urine, is presented in the loading plot (C).

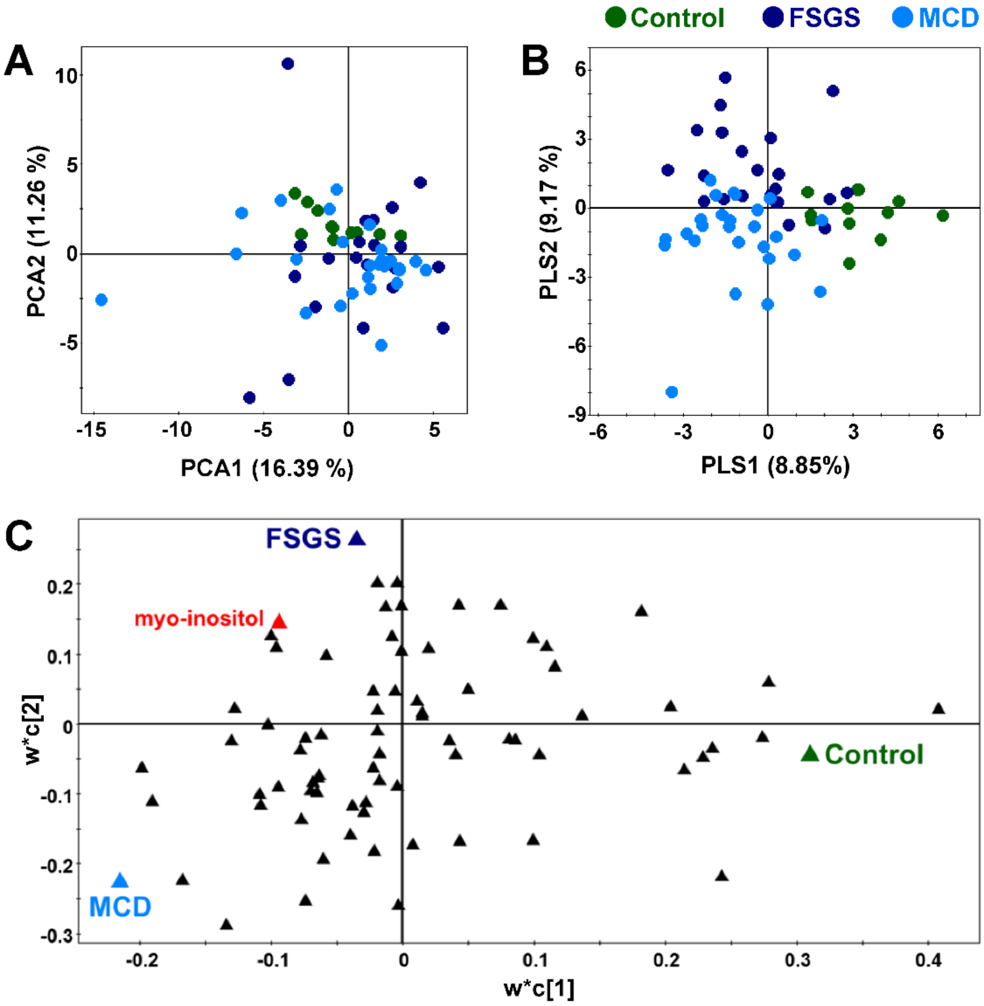

Figure S5. Full-length gels and blots, and multiple exposures of GAPDH (A), Synaptopodin (B), and ZO-1 (C)

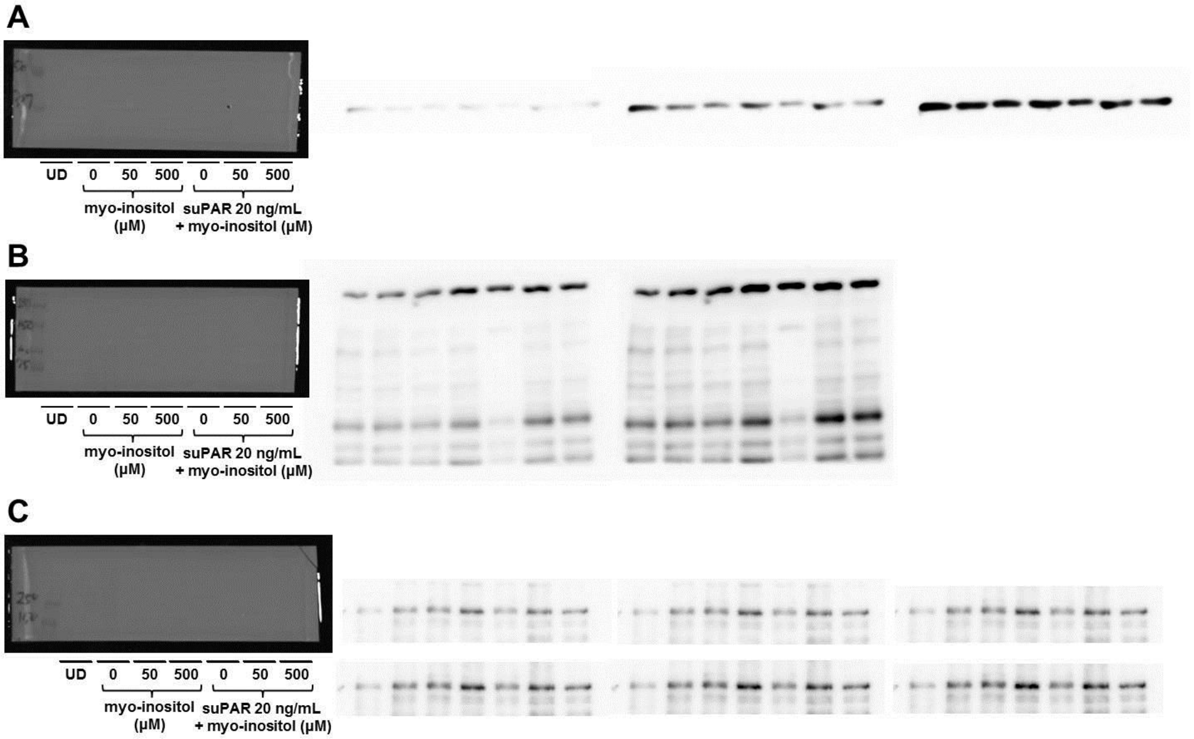

Figure S6. Subgroup analysis according to basal renal function. Log(urine myo-inositol) was higher in FSGS than in healthy control or MCD, regardless of whether eGFR was ≥ 60 ml/min/1.73 m^2, or < 60 ml/min/1.73 m^2. The discovery set and the validation set showed similar results.

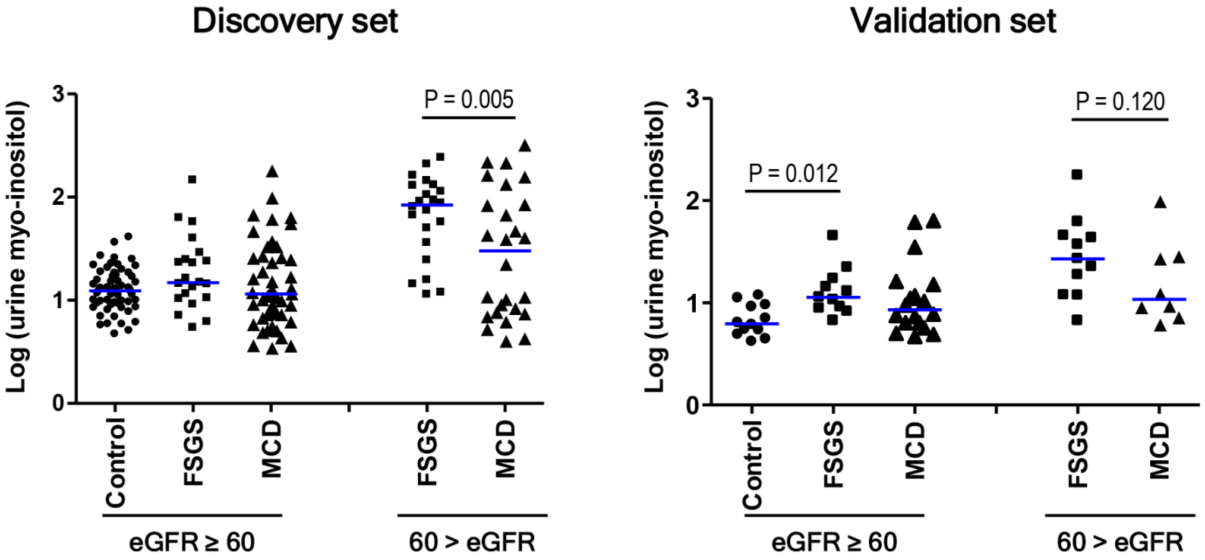

Figure S7. Enrollment of the discovery set and the validation set.

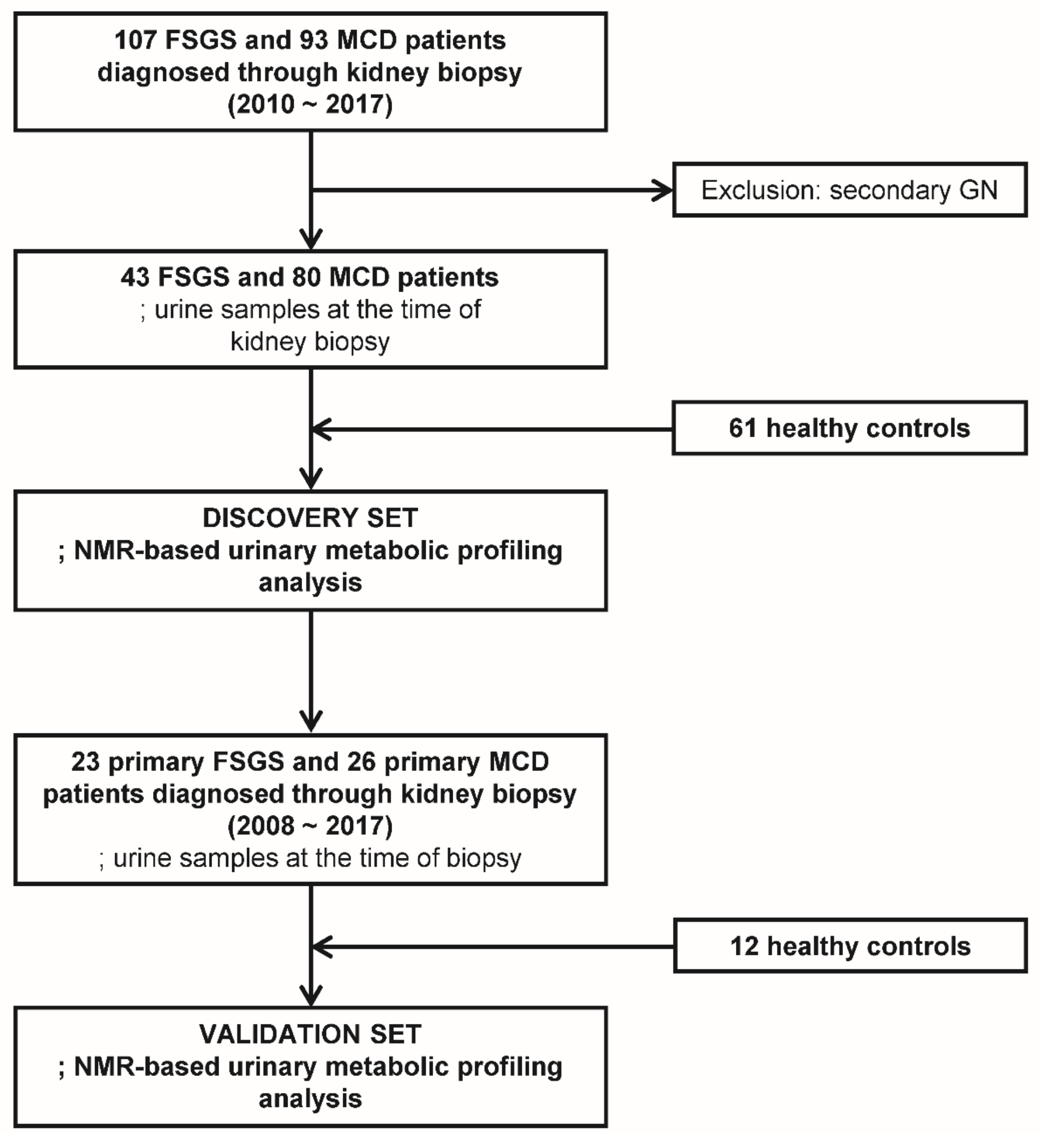

Supplement: Supplementary file 1 — Supplementary Information [file 41598_2019_51276_MOESM1_ESM.pdf]
